# Salvianolic acid B plays an anti-obesity role in high fat diet-induced obese mice by regulating the expression of mRNA, circRNA, and lncRNA

**DOI:** 10.7717/peerj.6506

**Published:** 2019-02-28

**Authors:** Tian An, Jing Zhang, Bohan Lv, Yufei Liu, Jiangpinghao Huang, Juan Lian, Yanxiang Wu, Sihua Gao, Guangjian Jiang

**Affiliations:** 1 Traditional Chinese Medicine School, Beijing University of Chinese Medicine, Beijing, China; 2 Department of Endocrinology, Tangshan Workers Hospital, Tangshan, China; 3 The Third Affiliated Hospital, Beijing University of Chinese Medicine, Beijing, China; 4 University of Southern California, Los Angeles, CA, USA

**Keywords:** Sal B, Obesity, circRNA, Adipose tissue, lncRNAs

## Abstract

**Background:**

Adipose tissue plays a central role in obesity-related metabolic diseases such as type 2 diabetes. Salvianolic acid B (Sal B), a water-soluble ingredient derived from *Salvia miltiorrhiza*, has been shown to reduce obesity and obesity-related metabolic diseases by suppressing adipogenesis. However, the role of Sal B in white adipose tissue (WAT) is not yet clear.

**Methods:**

Illumina Hiseq 4000 was used to study the effects of Sal B on the expression of long non-coding RNA (lncRNA) and circular RNA (circRNA) in epididymal white adipose tissue induced by a high fat diet in obese mice.

**Results:**

RNA-Seq data showed that 234 lncRNAs, 19 circRNAs, and 132 mRNAs were differentially expressed in WAT under Sal B treatment. The up-regulated protein-coding genes in WAT of the Sal B-treated group were involved in the insulin resistance pathway, while the down-regulated genes mainly participated in the IL-17 signaling pathway. Other pathways may play an important role in the formation and differentiation of adipose tissue, such as B cell receptor signaling. Analysis of the lncRNA–mRNA network provides potential targets for lncRNAs in energy metabolism. We speculate that Sal B may serve as a potential therapeutic approach for obesity.

## Introduction

Obesity has become a worldwide problem and a major risk factor for diabetes, infertility, and cardiovascular disease (Hu et al., 2016; Liang et al., 2017; Ferramosca et al., 2016; Supriya et al., 2018). It is a chronic metabolic disease characterized by abnormal fat distribution or excessive lipid accumulation (Yang et al., 2012). White adipose tissue (WAT), the central metabolic organ that regulates the energy homeostasis of the body (Choe et al., 2016), is closely associated with the occurrence of obesity and related complications (Hajer, Van Haeften & Visseren, 2008).

Noncoding RNA (ncRNA) is a general term for all functional RNAs that are not translated into proteins. As biological mediators, they can participate in the regulation of gene expression through epigenetic modification, transcriptional regulation and post-transcriptional regulation (Tang, Chen & Zhou, 2018). With the development of sequencing technology, a variety of new non-coding RNAs have been discovered, and their role in gene regulatory networks and regulation of endothelial cell function and metabolism is becoming better understood (Santulli, 2015, 2016; Liu et al., 2018). Among them, circular RNA (circRNA) and long non-coding RNA (lncRNA) play roles in regulating beta cell function, influencing transformation of adipose tissue and its energy metabolism and making them a promising target for anti-obesity therapy (Kaur, Mirza & Pociot, 2018; Li et al., 2018; Zhu et al., 2019).

Salvianolic acid B (Sal B) is a water-soluble component extracted from the traditional Chinese medicinal plant *Salvia miltiorrhiza* (Family Labiatae; referred to herein as *S. miltiorrhiza*) (Wang et al., 2014). In recent years, studies have shown that Sal B can reduce obesity and obesity-related metabolic disorders (Chien et al., 2016; Pan et al., 2018). Our work has shown that Sal B can improve glycolipid metabolism and reduce body weight in obese mice induced by high fat diet (HFD) (Zhao et al., 2017). However, it is not known whether anti-obesity activity of Sal B is related to the regulation of non-coding RNA expression. In this study, we aimed to investigate the effect of Sal B on the expression of lncRNA and circRNA in epididymis white adipose tissue (EP) of obese mice induced by a HFD. The anti-obesity target of Sal B was screened using functional studies of differentially expressed lncRNA and mRNA.

## Materials and Methods

### Ethics statement

All the study protocol was approved by the Animal Care and Management Committee of the Beijing University of Chinese Medicine, and all animal manipulations were according to the guidelines of the Animal Care Committee.

### Mice experiment and tissue extraction

Male C57BL/6J mice provided by SPF (Beijing) Biotechnology Co. Ltd. (Certificate NO. SCXK (Jing) 2016-0002) were used in this study. After 1-week acclimation, the mice were fed a HFD (60% fat) for 12 weeks to induce obesity (average body weight: HFD > (1 + 20%) normal, *n* = 10). After that, obesity mice were randomly divided into EP-S and EP-M groups (*n* = 5). Sal B intervention group (EP-S) and model (EP-M) groups were respectively administered Sal B (75 mg/kg body weight/day) or vehicle (an equivalent volume of water) by oral gavage daily for 8 weeks. At the end of the study, blood samples and EPs were collected from all the mice sacrificed by cervical dislocation, and immediately frozen in liquid nitrogen and stored at −80 °C for subsequent analysis.

### Measurement of blood lipid profiles and body fat mass

The concentrations of serum total cholesterol (TC), triglyceride (TG), low-density lipoprotein cholesterol (LDL), and high-density lipoprotein cholesterol (HDL) were determined using chemistry reagent kits (Nanjing Jiancheng Biology Engineering Institute, Nanjing, China) and an automated biochemical analyzer (Hitachi, Tokyo, Japan). Body composition and total fat mass was measured by magnetic resonance imaging (EchoMRI-100 for mice; Echo Medical System, Houston, TX, USA) after the Sal B administration (weeks 8).

### RNA isolation and RNA-seq analysis

Total RNAs from tissues were extracted using the Trizol reagent and purified using RiboZero Magnetic Gold Kit according to the manufacturer’s instructions. RNA sequencing libraries were generated using the KAPA Stranded RNA-Seq Library Prep Kit. The constructed cDNA libraries were qualified by Agilent 2100 Bioanalyzer, quantified by qPCR, and sequenced on an Illumina Hiseq 4000.

### Functional enrichment analysis

Using Gene Ontology (GO) database (http://www.geneontology.org). We analysis the GO enrichment of the differentially expressed mRNAs and their functions, based on three aspects: biological processes (BP), cellular components (CC), and molecular functions (MF). The log 10 values (*p*-value) denote enrichment scores and represent the significance of the GO term enrichment among the differentially expressed genes. KEGG pathway analysis revealed pathway clusters covering the differentially expressed genes, and the log 10 values (*p*-value) denote the enrichment score and represent the significance of the pathway correlations. In addition, Gene Set Enrichment Analysis (GSEA) is used to compensate for the shortcomings of individual genes in analysis.

### Correlation and co-expression analysis of mRNA and lncRNA

Based on the inter-regulatory association between differentially expressed genes in the EP between Sal B treatment group and obesity group, the lncRNA–mRNA regulatory network was constructed using Cytoscape v2.8.2 software (http://www.cytoscape.org/).

### Quantitative real time-PCR

Quantitative real time (qRT)-PCR was performed to verify the results of RNA-seq. Total RNAs were isolated from samples using the Trizol reagent, then reverse-transcribed into cDNA according to the manufacturers’ instruction. The transcriptase reactions contained 1.5 μg RNA, 0.5 μg/μl random primers (N9, 1 μl), 2.5 mM dNTPs mix (1.6 μl), 5× first-strand buffer (4.0 μl), 0.1M dithiothreitol (1 μl), RNase inhibitor (0.3 μl), and Superscript III RT (0.2 μl). Sybr Green-based qPCR was performed using SYBR Premix ExTaq. All data were normalized to data for ARBP to calculate relative mRNA concentrations. The primers used in this study are shown in Table 1.

**Table 1:** Primers for quantitative PCR analysis.

Gene	Forward (5′–3′)
ARBP	F: TTTGGGCATCACCACGAAAA R: GGACACCCTCCAGAATTTTC
Wbscr27	F: TGAGCTCTTAAGAGTCACCAAG R: CTTGTTCTGATGTTGCATGCTC
Sfrp5	F: CAAGATGCGCATTAAGGAGATC R: CTTGAGCAGCTTCTTCTTC
Adig	F: TCACACTCTCTTTGGTTTT R: CCAGTTGAAGCACAAATCTGAA
chr7:67264864–67268400-	F: AGACCTCACGGTGCCAAAT R: CTTTCTTTCTTAACGTCCACAGG
Saa3	F: CAGTTCATGAAAGAAGCTGGTC R: CGAGCATGGAAGTATTTGTCTG
ENSMUST00000169194	F: GGCAGGCATGACTAAATG 3 R: CAGGGTTGATTAGCAGTGTC

### Statistical analysis

The statistical differences were analyzed using the SPSS (version 20.0; IBM SPSS Statistics, Chicago, IL, USA) by independent-samples *t*-test. All data were shown as the means ± SD. *p*-values < 0.05 were regarded as statistically significant.

## Results

### Effects of Sal B on body fat mass and serum lipid profiles of obese mice induced by HFD

After 8 weeks of Sal B intervention, the body weights, TG, TC, HDL, LDL, and body fat mass of experimental mice in the two groups were measured. The body weight, TG, TC, HDL, LDL, and body fat mass in the obesity group was significantly higher than that in Sal B treatment group (*p* < 0.05) (Table 2). These results indicate that Sal B can reduce the body weight and fat mass as well as prevent dyslipidemia caused by HFD feeding.

**Table 2:** Effects of Sal B on body fat mass and serum lipid profiles of obese mice induced by HFD.

Name	Obesity model group	Sal B treatment group
**TG**	0.984 ± 0.106	0.777 ± 0.069*
**TC**	6.229 ± 0.483	5.011 ± 0.391*
**LDL-C**	0.425 ± 0.021	0.317 ± 0.029*
**HDL-C**	1.213 ± 0.191	1.876 ± 0.105*
**Fat mass**	0.431 ± 0.011	0.356 ± 0.015*

**Notes:**

TC, total cholesterol; TG, triglyceride; LDL, low-density lipoprotein cholesterol; and HDL, high-density lipoprotein cholesterol.

*n* = 6, values are presented as mean ± SD. Significant differences by *p < 0.05.

### Effects of Sal B on mRNAs and circRNAs expression in EP of obese mice induced by HFD

**Figure 1:**
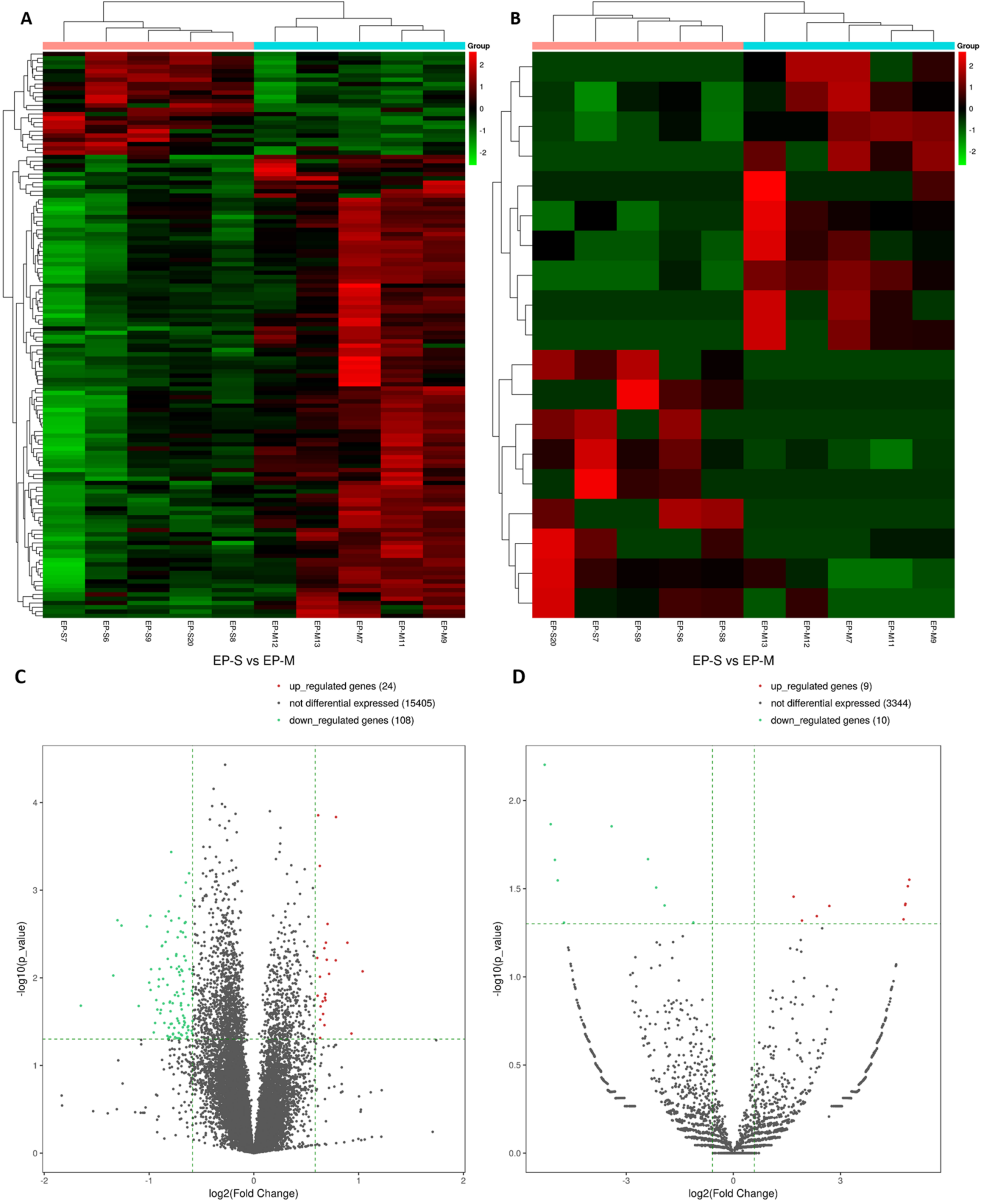
Analysis of DEmRNAs (A, C) and DEcircRNAs (B, D). Hierarchical clustering. Each row represents an mRNA and each column represents a sample. Green and red represent down-and up-regulated mRNAs or circRNAs, respectively. Genes in the volcano-Plot above the green parallel line (*p* < 0.05) and outside the two longitudinal green lines indicated DEmRNAs and DEcircRNAs between the two compared samples.

In total, 15,184 mRNAs were identified, of which 132 differentially expressed (DEmRNAs). In the EP-S group, 24 were up-regulated and 108 were down-regulated (Figs. 1A and 1C; Table S1). Compared with EP-M, there were 19 differentially expressed circRNAs in the EP-S, of which nine were up-regulated and 10 were down-regulated (Figs. 1B and 1D; Table S2). Among DEmRNAs, the up-regulated expression of Wbscr27 was the highest, with a fold change of 2.053, while C1rb was the most down-regulated, with a fold change of 0.318. In addition, some mRNAs that have been shown to play a role in fat metabolism, such as Sfrp5, Adig, and Saa3 were also differentially expressed.

### Effects of Sal B on the expression and function of lncRNAs in EP induced by HFD in obese mice

**Figure 2:**
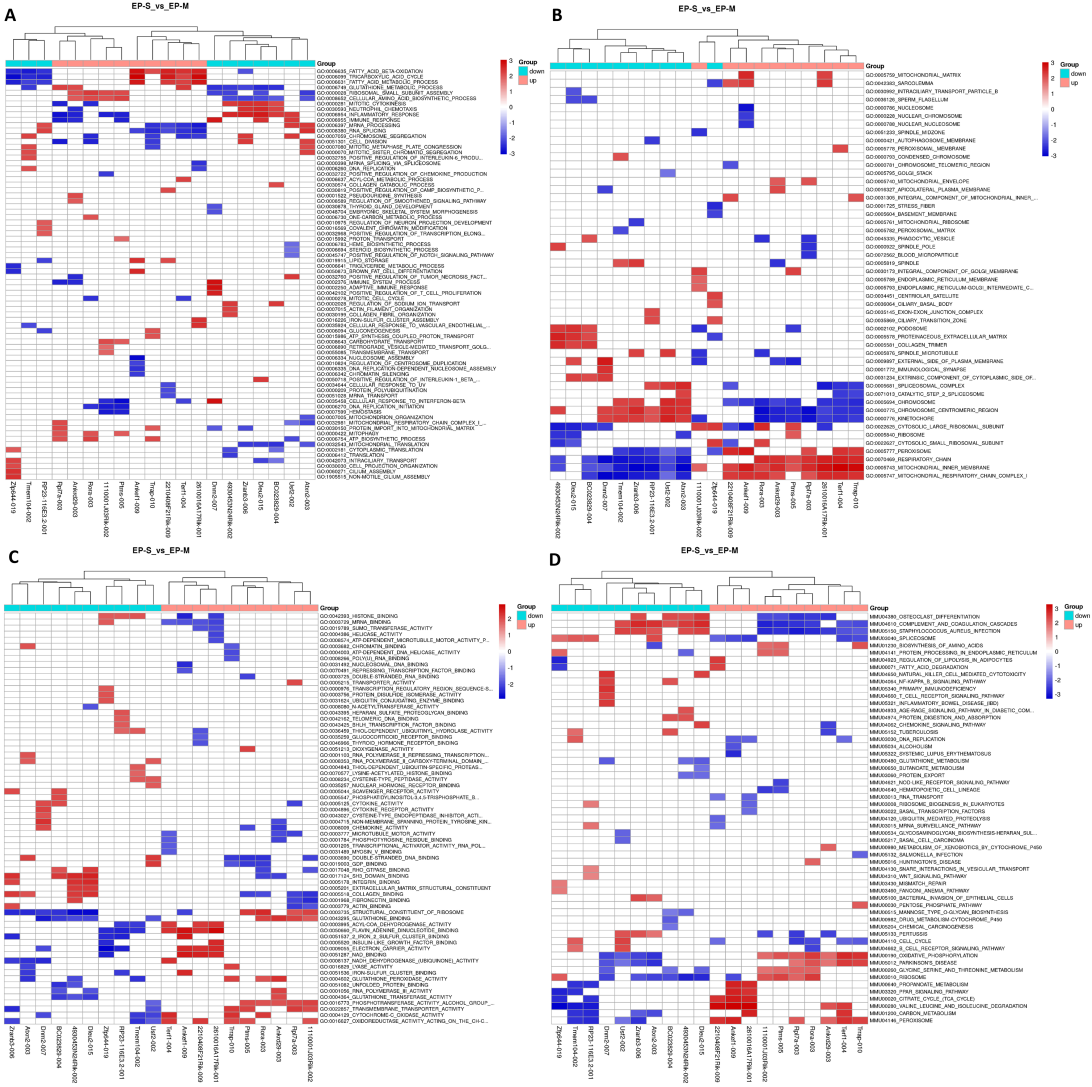
GSEA Cluster Heat Map of top 10 DElncRNAs, in up-regulation and down-regulation, respectively. (A) Biological process; (B) cellular components; (C) molecular functions, and (D) KEGG pathway, each row represents a functional entry, and each column represents an lncRNA. GSEA is a method used to determine whether a given gene set has significant differences among different groups. Genes in these sets have some degree of correlation. Therefore, enrichment analysis of gene sets can make up for the shortcomings of single gene in the analysis.

We found 234 differentially expressed lncRNAs (DE lncRNAs), including 87 up-regulated and 147 down-regulated in the experimental group (Table S3). Based on this, we performed a GSEA functional analysis of the DElncRNAs, and found that the up-regulated expression of lncRNAs are mainly involved in brown adipocyte differentiation, steroid biosynthesis, lipid transport, and lipid metabolism, while the down-regulated expression of lncRNAs are associated with the immune process and inflammatory responses (Fig. 2). After classifying the DElncRNAs, 179 were found to be exon sense overlapping, 11 were intergenic, 17 were intron sense overlapping, 16 were antisense, and 11 were bidirectional.

### qRT-PCR validation of differentially expressed mRNAs, lncRNAs, and circRNAs

**Figure 3:**
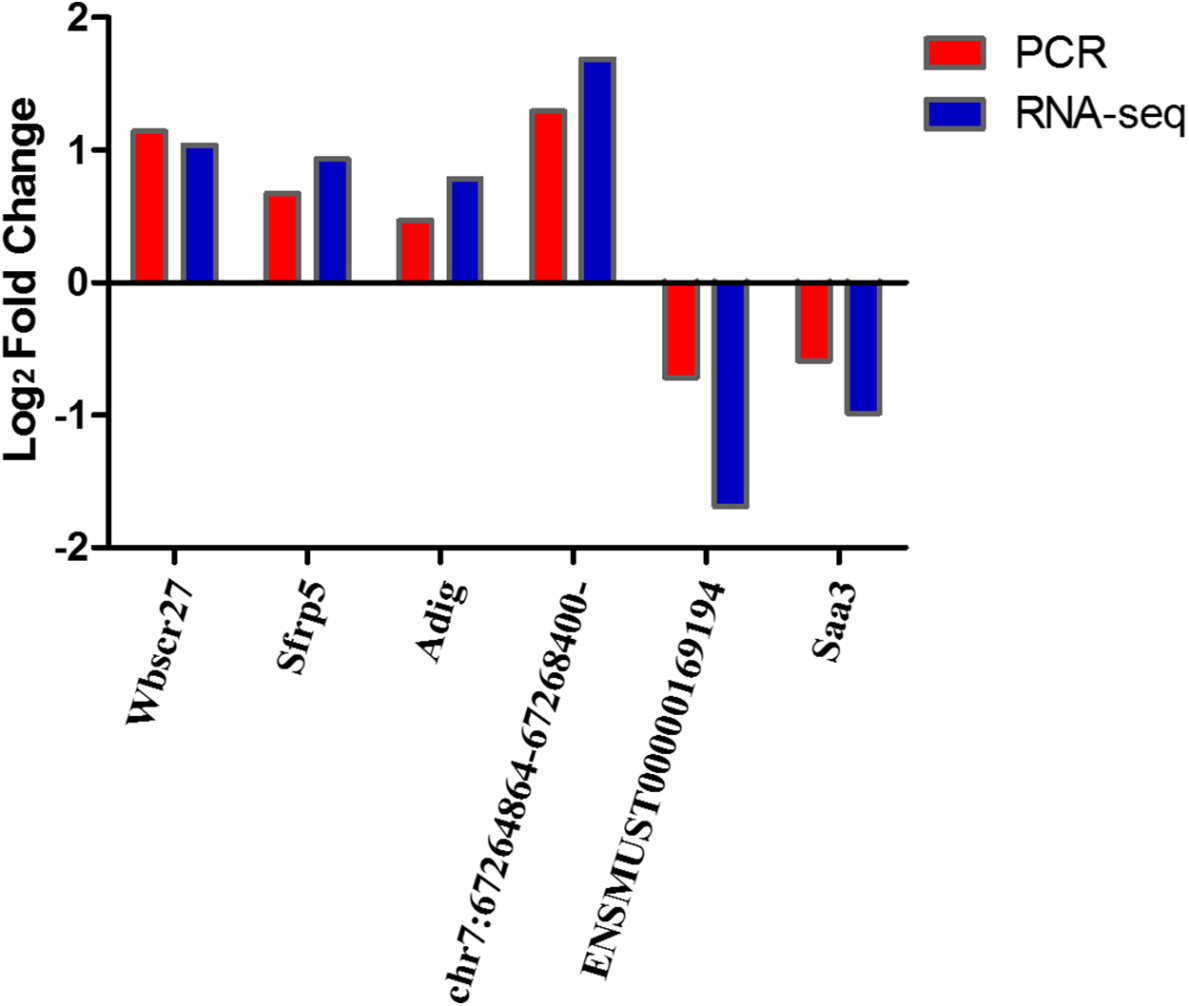
Sequencing and quantitative PCR. Sequencing and quantitative PCR for mRNAs (Wbscr27, Sfrp5, Adig, and Saa3), circRNAs- chr7:67264864–67268400:- and lncRNA-ENSMUST00000169194. The quantitative PCR results were consistent with the sequencing data. *n* = 5.

We selected four DEmRNAs (Wbscr27, Sfrp5, Adig, and Saa3), DEcircRNAs- chr7:67264864–67268400:- and DElncRNA-ENSMUST00000169194, which are most relevant to obesity, for use in verifying RNA-seq results using qRT-PCR. Results showed that the expression levels of Wbscr27, Sfrp5, Adig, and chr7:67264864–67268400:- were up-regulated in the EP-S group compared with the EP-M group, consistent with the sequencing results. The expressions of ENSMUST00000169194 and Saa3 were down-regulated in the EP-S group, which was also consistent with the sequencing results (Fig. 3).

### LncRNA–mRNA regulatory analysis

**Figure 4:**
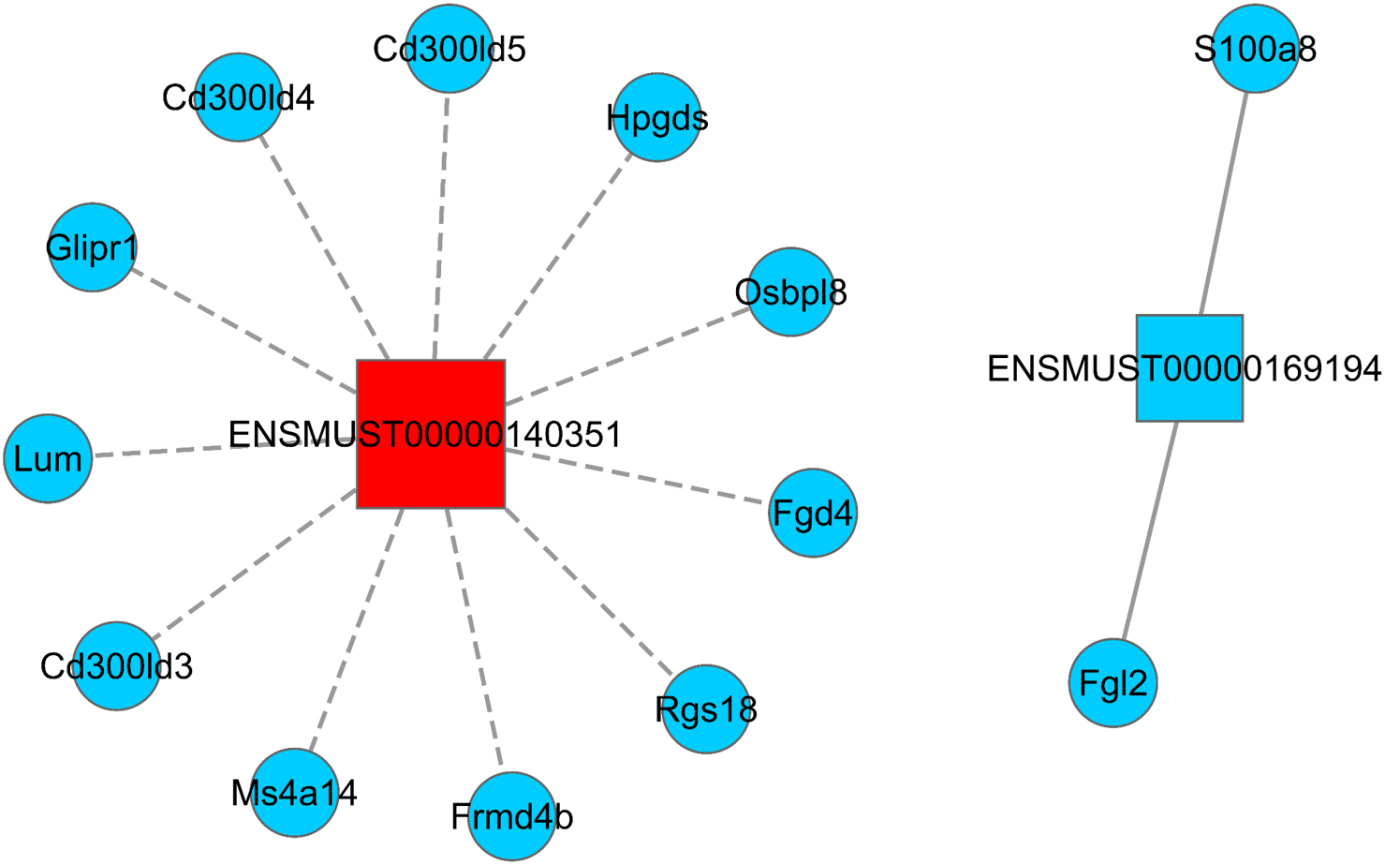
LncRNA–mRNA regulatory network (ENSMUST0000140351 and ENSMUST00000169194). Squares represent lncRNAs, circles represent mRNAs; red indicates up-regulated expression and blue indicates down-regulated expression. The solid line is positively correlated and the dotted line is negatively correlated. LncRNA–mRNA regulatory network was constructed using Cytoscape v2.8.2 software.

LncRNA has the ability to regulate several groups of mRNAs at the transcriptional level, including positive and negative regulation. Hence, understanding how Sal B alters the expression of lncRNA and its target mRNA expression is key to understanding its molecular mechanism of anti-obesity. We screened two DElncRNAs (ENSMUST0000140351 and ENSMUST00000169194) to construct the lncRNA–mRNA regulatory network map. A total of 11 differentially expressed mRNAs were found to be negatively correlated with the expression of ENSMUST0000140351 (Ms4a14, Cd300ld3, Lum, Glipr1, Cd300ld5, Rgs18, Cd300ld4, Fgd4, Hpgds, Frmd4b, and Osbpl8). We also found that the expressions of S100a8 and Fgl2 were positively correlated with the expression of ENSMUST00000169194 (Fig. 4; Table S4).

### Functional analysis of DEmRNAs

**Figure 5:**
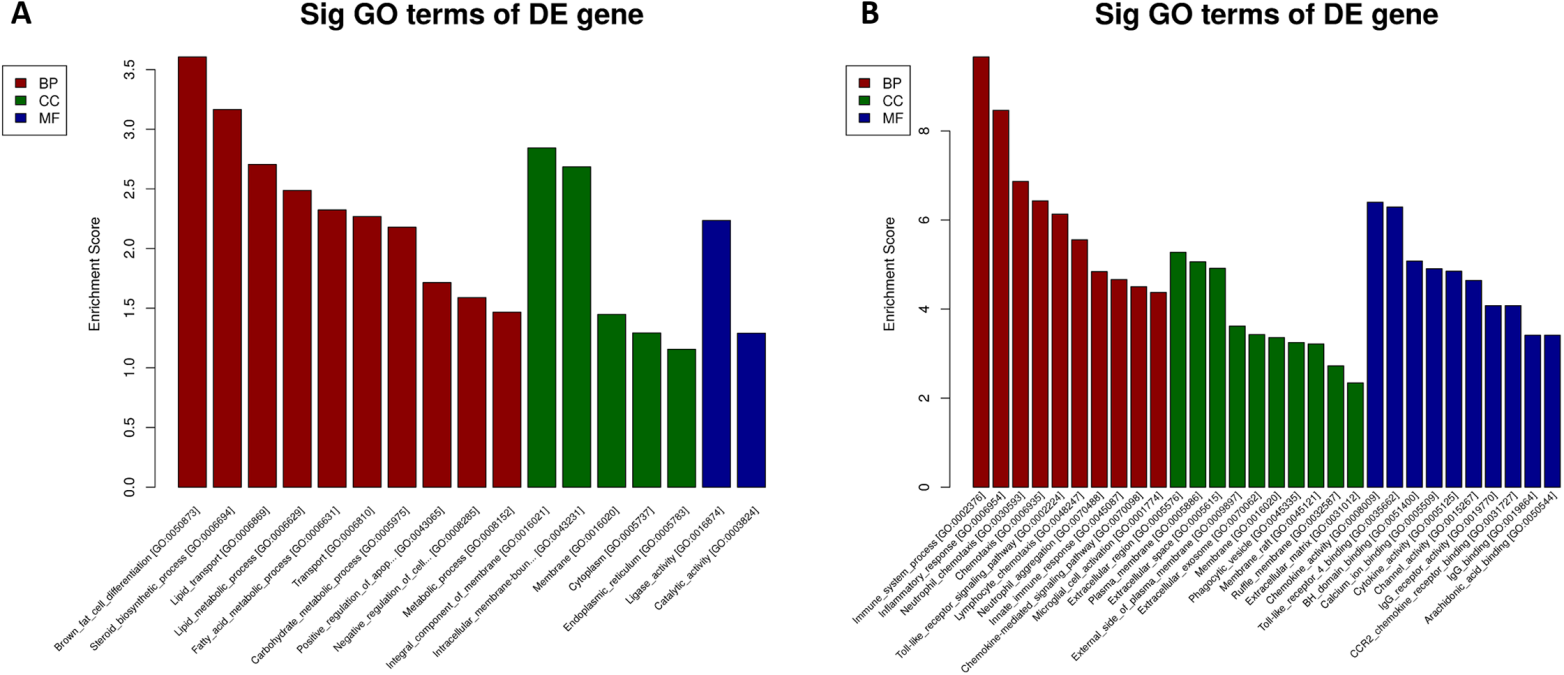
GO analysis. (A) up-regulated and (B) down-regulated of DEmRNAs. Using GO database (http://www.geneontology.org) analysis the GO enrichment of the DEmRNAs, based on three aspects: biological processes (BP), cellular components (CC), and molecular functions (MF). The log 10 values (*p*-value) denote enrichment scores and represent the significance of the GO term enrichment among the DEmRNAs.

We performed GO and KEGG enrichment analysis to determine the functional significance of DEmRNAs in the EP-S/EP-M group. GO enrichment analysis showed that up-regulated mRNA was enriched in 13 BP, 5 CC, and 2 MF, while down-regulated mRNA was enriched in 89 BP, 24 CC, and 35 MF (Fig. 5; Table S5). The most highly enriched up-regulated GO terms were “brown fat cell differentiation (biological process),” “integral component of membrane (CC),” and “ligase activity (MF),” while the most highly enriched down-regulated GO terms were “immune system process (BP),” “extracellular region (CC),” and “chemokine activity (MF).”

KEGG pathway analysis showed that differentially expressed mRNAs were enriched in 14 pathways associated with obesity (*p* < 0.05). The most highly enriched pathways were “insulin resistance” and “IL-17 signaling pathway.” Notably, we also identified mRNAs involved in the regulation of these pathways, which may provide targets for Sal B as a potential drug to prevent HFD-induced obesity (Table 3). Moreover, a number of metabolic-related pathways were screened, including the “NF-κB signaling pathway” and the “B-cell receptor signaling pathway.” KEGG pathway analysis suggests the possibility that Sal B exerts its weight reduction effect through the regulation of adipose metabolism via insulin resistance and IL-17 signaling in HFD induced-obesity mice.

**Table 3:** KEGG pathway analysis.

ID	Term	Count	Genes
**mmu04931**	Insulin resistance	2	Slc27a1, Slc2a4
**mmu04657**	IL-17 signaling pathway	6	Ccl12, Ccl7, Cxcl1, Fosl1, S100a8, S100a9
**mmu04145**	Phagosome	7	Atp6v0d2, Comp, Cybb, Fcgr1, Fcgr4, Msr1, Rab7b
**mmu05323**	Rheumatoid arthritis	5	Atp6v0d2, Ccl12, Ccl3, Cd86, Il18
**mmu04064**	NF-kappa B signaling pathway	5	Bcl2a1a, Bcl2a1b, Bcl2a1d, Btk, Card11
**mmu04380**	Osteoclast differentiation	5	Btk, Fcgr1, Fcgr4, Fosl1, Lilra5
**mmu04662**	B cell receptor signaling pathway	4	Btk, Card11, Cd72, Rasgrp3
**mmu04620**	Toll-like receptor signaling pathway	4	Ccl3, Cd86, Tlr1, Tlr8
**mmu04060**	Cytokine-cytokine receptor interaction	6	Ccl12, Ccl3, Ccl7, Cxcl1, Cxcl16, Il18
**mmu04062**	Chemokine signaling pathway	5	Ccl12, Ccl3, Ccl7, Cxcl1, Cxcl16
**mmu04621**	NOD-like receptor signaling pathway	4	Ccl12, Cxcl1, Cybb, Il18
**mmu04668**	TNF signaling pathway	3	Ccl12, Cxcl1, Gm5431
**mmu04210**	Apoptosis	3	Bcl2a1a, Bcl2a1b, Bcl2a1d
**mmu04933**	AGE–RAGE signaling pathway	2	Ccl12, Cybb

## Discussion

Epigenetic alteration refers to a heritable change in gene expression under conditions in which the genomic DNA sequence does not change, resulting in an altered phenotype. This includes changes in the expression of non-coding RNA (Takada, Kouzmenko & Kato, 2009). Studies have shown that epigenetic modification plays an important role in the occurrence and development of obesity (Kasinska, Drzewoski & Sliwinska, 2016; Huang et al., 2018). With the advancement of RNA sequencing technology, more and more non-coding RNAs related to energy metabolism are recognized as involved in obesity and related metabolic diseases. WAT is mainly distributed in the subcutaneous tissue, omentum and mesentery of mice, with epididymis white fat (EP) as the most commonly used WAT in adipose studies. We studied the effects of Sal B on the expression of mRNAs, lncRNAs, and circRNAs in EP of HFD induced obesity mice from an epigenetic level, and explored the anti-obesity effect of Sal B.

Obesity is an inflammatory state that occurs in adipose tissue, and therefore constitutes a chronic inflammatory disease accompanied by activation of inflammatory signaling pathways in adipose tissue cells, release of inflammatory cytokines, and infiltration of immune cells (Nteeba et al., 2013; Mathieu, Lemieux & Després, 2010). Therefore, research on the treatment of obesity inflammation and new target exploitation will provide novel targets for the treatment of obesity and its related metabolic diseases. In the present study, we found that the expression of many inflammation-associated mRNAs was affected by Sal B treatment, including Sfrp5 and Saa3. Secreted frizzled-related protein-5 (Sfrp5), known as an anti-inflammatory adipokine, is much more abundant in adipose tissue than other tissues, and negatively affects obesity and obesity-related metabolic disorders (Hu et al., 2013). Previous studies have shown that in the adipose tissue of Sfrp5 knockout mice, the number of macrophages is significantly increased, and the expression of factors related to cellular inflammatory activity, such as TNF-a and IL-6, are significantly increased (Ouchi et al., 2010). Sfrp5 exerts anti-inflammatory effects by binding to Wnt5a to inhibit the activation of the downstream target JNK of the Wnt pathway and reduce the secretion of inflammatory factors in obese mice (Catalán et al., 2014) as well as in 3T3-L1 cells (Shadid & Jensen, 2003). Consistent with previous research, we found that Sfrp5 is up-regulated 1.9-fold under Sal B treatment. Therefore, we hypothesize that Sal B may exert anti-obesity effects by regulating the expression of inflammation-related mRNA in adipose tissue. In addition, Sfrp5 can be used as a candidate target for studying the anti-obesity mechanism of Sal B, and its specific mechanism should be the emphasis of future research.

Another inflammation-related mRNA, Saa3, was found to be expressed at half the control rate under Sal B treatment, consistent with previous studies. The serum amyloid A family is a class of proteins released during acute inflammatory response and is closely related to the pathogenesis of chronic inflammatory diseases such as obesity (Van Dielen et al., 2001). The expression of Saa3 is significantly increased under a promoted adipocyte inflammatory response by saturated fatty acids and glucose. In addition, Saa3 is highly expressed in the adipose tissue of obese mice, which may be related to the induction of adipose tissue inflammation (Den Hartigh et al., 2014). In this study, we found that the expression of Saa3 was significantly down-regulated in EP-S, suggesting that Sal B can reduce the inflammatory response induced by Saa3.

In the EP-M group, we found some abnormal expression of mRNA associated with adipose transformation, with Sal B intervention reversing these changes. Adipoietin (Adig), also known as small adipocytokines 1, plays a significant role in the differentiation of adipocytes (Ren et al., 2016). Our results showed that the expression Adig in the Sal B treatment group was significantly higher than in the obese model group. This is in line with the previous findings that Adig can promote the differentiation of adipocytes by activating the expression of PPARγ, Srebp-1, and Fas genes (Mei, Zhang & Fu, 2016). Therefore, we speculate that Sal B can up-regulate the expression level of Adig to activate the adipose transcription factor and promote the differentiation of adipocytes.

We identified 234 DElncRNAs, including 87 up-regulations and 147 down-regulations. Several differentially expressed lncRNAs might participate in lipid metabolism and glucose metabolism. For example, Rora and Dnm2 were predicted to be involved in “oxidative phosphorylation” and “glycine, serine, and threonine metabolism.” This is consistent with previous studies showing that Rora regulates lipid metabolism (Kim et al., 2017). Therefore, the differential expression profiles obtained indicate that Sal B can exert a potential regulatory function in fat deposition and metabolism in obese mice by modulating the expression of lncRNAs.

Gene Ontology and KEGG pathway analyses were performed to predict the possible functions of DEmRNAs. Our results indicated that the up-regulated expression of mRNAs involved in BP is primarily associated with brown adipocyte differentiation (Adig and Slc2a4), lipid metabolic process (Aacs, Fdx1, and Slc27a1), and metabolic process (Aacs and Slc27a1). The down-regulated mRNAs might be related to inflammatory response (Ccl12, Ccl3, Ccl7, Cd180, Cxcl1, Cybb, Il18, Ly86, S100a8, S100a9, Tlr1, and Tlr8). KEGG pathway analysis showed that two DEmRNAs, Slc27a1 and Slc2a4, were involved in the insulin resistance signaling pathway, which is closely related to obesity (Yu, Kim & Lee, 2017). The inflammatory signaling, IL-17 signaling, and NF-kappa B signaling pathways were also subjected to KEGG annotation prediction analysis (Tanti et al., 2012, Tarantino et al., 2014). Our results indicate that Sal B may exert anti-obesity effects by modulating the expression of mRNAs in lipid metabolism and inflammation-related signaling pathways.

Among all RNAs, circRNAs are the least understood, but appear to have complex regulatory effects in the development of obesity. We identified 9 up-regulated and 10 down-regulated circRNAs under Sal B treatment. Among them, the expression changes of chr14:103252408–103276518:- and chr14:103282597–103291362:- were the most obvious, with an approximate 30-fold upregulation. Therefore, these circRNAs may serve as targets for the design of therapeutic drugs for obesity, pending study of their specific mechanisms.

## Conclusions

This study is the first comprehensive analysis of mRNA, lncRNA, and circRNA expression in EP of HFD-induced obese mice. We found that Sal B regulates the expression of mRNAs and lncRNAs associated with adipocyte differentiation, lipid metabolism, and inflammation, as well as the insulin resistance and IL-17 signaling pathways. These findings suggest that Sal B plays an important role in inhibiting obesity by regulating anti-inflammatory related factors and signaling pathways. Our research provides valuable insights into the molecular mechanism of Sal B in anti-obesity effects and contributes therapeutic markers for pharmaceutical design in the prevention and treatment of obesity. In the future, the corresponding roles and molecular mechanisms of non-coding RNAs should be further elucidated. In addition, differential expression at the RNA level does not necessarily indicate that the expression of related proteins is also significantly different. We aim to correlate RNA and protein analysis to more fully investigate the anti-obesity mechanism of Sal B.

## Supplemental Information

10.7717/peerj.6506/supp-1 Supplemental Information 1 Differentially expressed mRNAs.

10.7717/peerj.6506/supp-2 Supplemental Information 2 Differentially expressed circRNAs.

10.7717/peerj.6506/supp-3 Supplemental Information 3 Differentially expressed lncRNAs.

10.7717/peerj.6506/supp-4 Supplemental Information 4 LncRNA–mRNA regulatory analysis data.

10.7717/peerj.6506/supp-5 Supplemental Information 5 GO enrichment analysis data.

## Abbreviations

LncRNAs: long non-coding RNAs
GO: Gene Ontology
WAT: white adipose tissue
Sal B: Salvianolic acid B
EP: epididymal white adipose tissue
HFD: high fat diet
circRNA: circular RNA
EP-S: Sal B intervention group
EP-M: obesity model group
TC: total cholesterol
TG: triglyceride
LDL: low-density lipoprotein cholesterol
HDL: high-density lipoprotein cholesterol
BP: biological processes
CC: cellular components
MF: molecular functions
GSEA: Gene Set Enrichment Analysis
DEmRNAs: differentially expressed mRNAs
DElncRNAs: differentially expressed lncRNAs
Sfrp5: Secreted frizzled-related protein-5
Saa: serum amyloid A.

## Additional Information and Declarations

### Data Availability

The following information was supplied regarding data availability: The raw measurements are available in Tables S1–S5.

## References

[ref-1] Catalán V, Gómez-Ambrosi J, Rodríguez A, Pérez-Hernández AI, Gurbindo J, Ramírez B, Méndez-Giménez L, Rotellar F, Valentí V, Moncada R, Martí P, Sola I, Silva C, Salvador J, Frühbeck G (2014). Activation of noncanonical Wnt signaling through WNT5A in visceral adipose tissue of obese subjects is related to inflammation. Journal of Clinical Endocrinology and Metabolism.

[ref-2] Chien M-Y, Ku Y-H, Chang J-M, Yang C-M, Chen C-H (2016). Effects of herbal mixture extracts on obesity in rats fed a high-fat diet. Journal of Food and Drug Analysis.

[ref-3] Choe SS, Jin YH, Hwang IJ, Kim JI, Kim JB (2016). Adipose tissue remodeling: its role in energy metabolism and metabolic disorders. Frontiers in Endocrinology.

[ref-4] Den Hartigh LJ, Wang S, Goodspeed L, Ding Y, Averill M, Subramanian S, Wietecha T, O’Brien KD, Chait A (2014). Deletion of serum amyloid A3 improves high fat high sucrose diet-induced adipose tissue inflammation and hyperlipidemia in female mice. PLOS ONE.

[ref-5] Ferramosca A, Conte A, Moscatelli N, Zara V (2016). A high-fat diet negatively affects rat sperm mitochondrial respiration. Andrology.

[ref-6] Hajer GR, Van Haeften TW, Visseren FLJ (2008). Adipose tissue dysfunction in obesity, diabetes, and vascular diseases. European Heart Journal.

[ref-7] Hu W, Li L, Yang M, Luo X, Ran W, Liu D, Xiong Z, Liu H, Yang G (2013). Circulating Sfrp5 is a signature of obesity-related metabolic disorders and is regulated by glucose and liraglutide in humans. Journal of Clinical Endocrinology & Metabolism.

[ref-8] Hu J, Yang S, Zhang A, Yang P, Cao X, Li X, Goswami R, Wang Y, Luo T, Liao K, Cheng Q, Xiao X, Li Q (2016). Abdominal obesity is more closely associated with diabetic kidney disease than general obesity. Diabetes Care.

[ref-9] Huang Q, Ma C, Chen L, Luo D, Chen R, Liang F (2018). Mechanistic insights into the interaction between transcription factors and epigenetic modifications and the contribution to the development of obesity. Frontiers in Endocrinology.

[ref-10] Kasinska MA, Drzewoski J, Sliwinska A (2016). Epigenetic modifications in adipose tissue–relation to obesity and diabetes. Archives of Medical Science.

[ref-11] Kaur S, Mirza A, Pociot F (2018). Cell type-selective expression of circular RNAs in human pancreatic islets. Non-Coding RNA.

[ref-12] Kim K, Boo K, Yu YS, Oh SK, Kim H, Jeon Y, Bhin J, Hwang D, Kim KI, Lee J-S, Im S-S, Yoon SG, Kim IY, Seong JK, Lee H, Fang S, Baek SH (2017). RORα controls hepatic lipid homeostasis via negative regulation of PPARγ transcriptional network. Nature Communications.

[ref-13] Li A, Huang W, Zhang X, Xie L, Miao X (2018). Identification and characterization of CircRNAs of two pig breeds as a new biomarker in metabolism-related diseases. Cellular Physiology and Biochemistry International Journal of Experimental Cellular Physiology Biochemistry and Pharmacology.

[ref-14] Liang P, Xi L, Shi J, Li W, Zhao S, Deng Y, Wang R, Sun Y, Gu B, Yuan L, Zhang Y, Gu W, Wang W, Hong J (2017). Prevalence of polycystic ovary syndrome in Chinese obese women of reproductive age with or without metabolic syndrome. Fertility and Sterility.

[ref-15] Liu H, Li H, Jin L, Li G, Hu S, Ning C, Guo J, Shuai S, Li X, Li M (2018). Long noncoding RNA GAS5 suppresses 3T3-L1 cells adipogenesis through miR-21a-5p/PTEN signal pathway. DNA and Cell Biology.

[ref-16] Mathieu P, Lemieux I, Després J-P (2010). Obesity, inflammation, and cardiovascular risk. Clinical Pharmacology & Therapeutics.

[ref-17] Mei C, Zhang Q, Fu C (2016). ADIG inducing the trans-differentiation of bovine myoblasts and its related gene expression. Xu Mu Yi Xue Bao.

[ref-18] Nteeba J, Ortinau LC, Perfield JW II, Keating AF (2013). Diet-induced obesity alters immune cell infiltration and expression of inflammatory cytokine genes in mouse ovarian and peri-ovarian adipose depot tissues. Molecular Reproduction and Development.

[ref-19] Ouchi N, Higuchi A, Ohashi K, Oshima Y, Gokce N, Shibata R, Akasaki Y, Shimono A, Walsh K (2010). Sfrp5 is an anti-inflammatory adipokine that modulates metabolic dysfunction in obesity. Science.

[ref-20] Pan Y, Zhao W, Zhao D, Wang C, Yu N, An T, Mo F, Liu J, Miao J, Lv B, Gu Y, Gao S, Jiang G (2018). Salvianolic acid B improves mitochondrial function in 3T3-L1 adipocytes through a pathway involving PPARγ coactivator-1α (PGC-1α). Frontiers in Pharmacology.

[ref-21] Ren G, Eskandari P, Wang S, Smas CM (2016). Expression, regulation and functional assessment of the 80 amino acid small adipocyte factor 1 (Smaf1) protein in adipocytes. Archives of Biochemistry and Biophysics.

[ref-22] Santulli G (2015). microRNAs distinctively regulate vascular smooth muscle and endothelial cells: functional implications in angiogenesis, atherosclerosis, and in-stent restenosis. Advances in Experimental Medicine and Biology.

[ref-23] Santulli G (2016). MicroRNAs and endothelial (Dys) function. Journal of Cellular Physiology.

[ref-24] Shadid S, Jensen MD (2003). Effects of pioglitazone versus diet and exercise on metabolic health and fat distribution in upper body obesity. Diabetes Care.

[ref-25] Supriya R, Tam BT, Yu AP, Lee PH, Lai CW, Cheng KK, Yau SY, Chan LW, Yung BY, Sheridan S, Siu PM (2018). Adipokines demonstrate the interacting influence of central obesity with other cardiometabolic risk factors of metabolic syndrome in Hong Kong Chinese adults. PLOS ONE.

[ref-26] Takada I, Kouzmenko AP, Kato S (2009). Wnt and PPARγ signaling in osteoblastogenesis and adipogenesis. Nature Reviews Rheumatology.

[ref-27] Tang H, Chen Y, Zhou H (2018). Research progress of long noncoding RNA in regulating adipogenesis. Zhong Nan Da Xue Xue Bao Yi Xue Ban.

[ref-28] Tanti JF, Ceppo F, Jager J, Berthou F (2012). Implication of inflammatory signaling pathways in obesity-induced insulin resistance. Frontiers in Endocrinology.

[ref-29] Tarantino G, Costantini S, Finelli C, Capone F, Guerriero E, La Sala N, Gioia S, Castello G (2014). Is serum Interleukin-17 associated with early atherosclerosis in obese patients?. Journal of Translational Medicine.

[ref-30] Van Dielen FMH, Van’t Veer C, Schols AM, Soeters PB, Buurman WA, Greve JWM (2001). Increased leptin concentrations correlate with increased concentrations of inflammatory markers in morbidly obese individuals. International Journal of Obesity and Related Metabolic Disorders Journal of the International Association for the Study of Obesity.

[ref-31] Wang P, Xu S, Li W, Wang F, Yang Z, Jiang L, Wang Q, Huang M, Zhou P (2014). Salvianolic acid B inhibited PPARγ expression and attenuated weight gain in mice with high-fat diet-induced obesity. Cellular Physiology and Biochemistry.

[ref-32] Yang Q, Cogswell ME, Flanders WD, Hong Y, Zhang Z, Loustalot F, Gillespie C, Merritt R, Hu FB (2012). Trends in cardiovascular health metrics and associations with all-cause and CVD mortality among US adults. JAMA.

[ref-33] Yu MK, Kim F, Lee WJ (2017). Role of NO/VASP signaling pathway against obesity-related inflammation and insulin resistance. Diabetes and Metabolism Journal.

[ref-34] Zhao D, Zuo J, Yu N, Fang X, Mo F, Wu R, Tian T, Ma R, Gao Y, Zhang D, Jiang G, Gao S (2017). Salvianolic acid B improves glucolipid metabolism by regulating adipogenic transcription factors in mice with diet-induced obesity. Journal of Traditional Chinese Medical Sciences.

[ref-35] Zhu E, Zhang J, Li Y, Yuan H, Zhou J, Wang B (2019). Long noncoding RNA Plnc1 controls adipocyte differentiation by regulating peroxisome proliferator-activated receptor γ. FASEB Journal.

