# Supplementary material for: Salvianolic acid B plays an anti-obesity role in high fat diet-induced obese mice by regulating the expression of mRNA, circRNA, and lncRNA
Source: PeerJ. 2019 Feb 28;7:e6506. doi: 10.7717/peerj.6506 (PMC6397762; doi:10.7717/peerj.6506)
Supplement: Supplemental Information 2 [file peerj-07-6506-s002.docx]

Supplement Table 2. Differentially expressed circRNA.

| **circRNA_ID** | **Gene_Name** | **Length** | **log2FC** | **Fold_Change** | **p** |
| --- | --- | --- | --- | --- | --- |
| **Up-regulated** |  |  |  |  |  |
| chr14:103252408-103276518:- | Mycbp2 | 676 | 4.928 | 30.432 | 0.028 |
| chr14:103282597-103291362:- | Mycbp2 | 1024 | 4.887 | 29.590 | 0.031 |
| mmu_circ_0001103 | Nfatc2 | 1208 | 4.820 | 28.253 | 0.039 |
| chr4:155527412-155543193:+ | Gnb1 | 470 | 4.811 | 28.074 | 0.039 |
| chr11:83501114-83502852:+ | Taf15 | 223 | 4.767 | 27.227 | 0.047 |
| chr15:3327973-3458008:- | Ghr | 820 | 2.688 | 6.446 | 0.040 |
| chr9:32129569-32153124:+ | Arhgap32 | 286 | 2.338 | 5.056 | 0.045 |
| mmu_circ_0000277 | Fxr2 | 825 | 1.922 | 3.788 | 0.048 |
| chr7:67264864-67268400:- | Mef2a | 412 | 1.687 | 3.219 | 0.035 |
| **Down-regulated** |  |  |  |  |  |
| chr2:104776171-104780260:- | Qser1 | 795 | -5.285 | 0.026 | 0.006 |
| mmu_circ_0000215 | Cpsf6 | 1599 | -5.115 | 0.029 | 0.014 |
| chr5:143092994-143098505:- | Rnf216 | 258 | -4.997 | 0.031 | 0.022 |
| chr3:100578475-100656274:- | Man1a2 | 1491 | -4.918 | 0.033 | 0.028 |
| chr12:69603475-69610826:- | Sos2 | 610 | -4.742 | 0.037 | 0.049 |
| chr5:28358273-28362234:+ | Rbm33 | 498 | -3.408 | 0.094 | 0.014 |
| chr7:99942958-99955540:- | Rnf169 | 221 | -2.389 | 0.191 | 0.022 |
| mmu_circ_0000375 | Hectd1 | 380 | -2.161 | 0.224 | 0.031 |
| chr4:59514273-59524476:- | Ptbp3 | 399 | -1.926 | 0.263 | 0.039 |
| chr3:109525868-109528384:+ | Vav3 | 329 | -1.118 | 0.461 | 0.049 |
